# “You treat what you have to treat, and you don’t care as much if they understand or if they feel good about it”: Communication barriers and perceptions of moral distress among doctors in emergency departments

**DOI:** 10.1097/MD.0000000000036610

**Published:** 2023-12-15

**Authors:** Clara Brune, Ann Liljas

**Affiliations:** a Karolinska Institutet, Department of Global Public Health, Stockholm, Sweden.

**Keywords:** dementia, doctors, emergency medicine, language barriers, moral distress

## Abstract

Doctors facing communication barriers when assessing patients in emergency departments (ED) is a frequent phenomenon, as the global prevalence of dementia and migration have increased. This study aims to explore how communication barriers influence moral distress as perceived by medical doctors working at emergency departments. Twelve doctors at 2 different EDs in Stockholm, Sweden, participated. Answers on communication barriers were collected from an interview guide on moral distress. Informants’ responses were analyzed using qualitative thematic analysis. The results suggest that doctors experience moral distress when assessing patients with communication barriers due to an inability to mediate calm and safety and understand their patients, and due an increased need of resources and difficulties in obtaining consent before conducting examinations or interventions. In conclusion, communication barriers can be a cause of moral distress, which should be considered when developing tools and methods to mitigate and manage moral distress.

## 1. Introduction

Functioning communication is crucial when assessing patients, as information from the patient including symptoms and medical history may determine treatment decisions. Particularly in the emergency department (ED), lacking information and delayed or missed examinations and treatments may cause acute health consequences for the patient, as the conditions causing the ED visit generally need rapid assessment. Meanwhile, doctors facing communication barriers as patients lack proficiency in the country spoken language or live with cognitive impairments such as dementia, are not uncommon in EDs.^[1–3]^ This is not surprising, as the global prevalence of dementia has increased substantially and is expected to continue to increase at an even higher rate.^[4]^ Similarly, migration has increased globally, and with it an increase in non-native speakers in many countries.^[5]^

Communication barriers impose risks for the patients. The ED itself can further complicate communication due to limited time, stress, lacking privacy and the often-chaotic environment.^[6]^ Studies on older adults’ perspectives of ED visits have identified poor communication as a problematic aspect of emergency care.^[7–9]^ Further, that research exploring ED communication with PLwD is sparse.^[8]^ A scoping review of communication between healthcare workers in EDs and patients living with dementia (PLwD) found that the dialogue was characterized by rushed communication, inconsistent information, and limited engagement in decision-making. This constitutes a common issue as PLwD visit the ED more frequently than patients without dementia.^[3]^ In fact, nearly half of all PLwD will visit the ED in any given year, and ED visits by PLwD are associated with adverse outcomes.^[3,10,11]^ One factor that has been attributed to this is poor communication.^[11]^

Language barriers in healthcare have been found to result in miscommunication between the medical professional and patient, and to reduce patient safety as well as the quality of care.^[12]^ One study found that the impact of language barriers on patients included medical errors, low treatment adherence and behavior, additional treatment cost and increased length of hospital stay. For healthcare providers, language barriers were found to affect their ability to take patient history, diagnose the patient, provide treatment, and resulted in an increased work burden.^[13]^ In Denmark, a country with fewer foreign-born compared to Sweden,^[14]^ a study found that language barriers frequently affected communication, which in turn negatively affected the clinical assessment and certainty of decision-making.^[2]^

Language barriers and communication barriers are known to increase the risk for patients and cause an increased work burden and moral distress for the healthcare provider.^[13,15–18]^ Moral distress describes the feelings of unease that arise in situations when someone knows the morally correct action to take but is constrained in some way from taking this action. It is related to malpractice, reduced quality of care, and poor communication, negatively affecting the patient.^[18,19]^ For doctors, moral distress is related to mental illness and decreased work motivation.^[19]^ In a nationwide survey on moral distress among doctors in the United Kingdom during the Covid-19 pandemic, 59.6% reported having experienced moral distress prior to the pandemic. Still, the concept seems to be unknown to many as 43.8% reported not having heard of moral distress. In the mentioned study, risk factors of experiencing moral distress included doctors being younger, belonging to ethnic minorities and doctors with disabilities. The study found that insufficient staffing was the main cause of moral distress.^[19]^ One of the obstacles that emergency medicine staff frequently face in their work and that may cause moral distress, are communication barriers. However, in what way communication barriers affect moral distress has not been researched. The aim of this study is to investigate doctors’ experiences of patient encounters involving communication barriers in EDs, with a focus on their experience of moral distress associated with these encounters.

## 2. Materials and methods

### 2.1. Study design and setting

This was a qualitative study, performed through semi-structured interviews. The study was conducted in Stockholm, the capital of Sweden. Twelve medical doctors were interviewed about their experiences of moral distress during the Covid-19 pandemic. The doctors worked at 2 hospital emergency departments (ED), purposively sampled to allow for 1 hospital in central Stockholm and 1 outside central Stockholm. The study sample of twelve interviews was decided as saturation was reached at this point. Saturation was deemed reached as no new codes were developed during the analysis of data, in line with the the definition of the concept by Urquhart, 2022.^[20]^

### 2.2. Participants

Managers at the 2 EDs, known to the principal researcher, distributed information about the study via posters and emails to all doctors working at the EDs. Doctors who expressed an interest in participating received written information about the study and their rights to withdraw, as well as the phone number and email address of principal researcher CB. Inclusion criteria were doctors who were currently employed at the EDs, and who had worked during the Covid-19 pandemic. Those meeting the interested in participating were invited to an interview at a time convenient to them. Participants were invited to ask questions about the study and their participation during the arrangements of the interview and before the start of the interview. This study was performed as a graduate student degree project. Studies undertaken by students do not require ethical approval according to Swedish law on ethical review (2003:460 2§). The study was still conducted in accordance with national ethical standards including participants’ rights to information, withdrawal and anonymity throughout the project. All informants gave formal consent to participate in the study in writing or verbally recorded before the interview started.

### 2.3. Interview questions

The interview guide contained questions in Swedish on the informants’ experiences of moral distress during and prior to the Covid-19 pandemic since this study was conducted as part of a larger study. Two out of 26 questions referred to whether language barriers and communication barriers affected the moral distress they experienced in their work:

1. Do you experience increased moral distress if the patient does not speak Swedish as a native language?
2. Have you experienced any other communication barriers, such as patient cognitive impairments, to affect your perception of moral distress? If yes, how?

Follow-up questions were asked if necessary for clarification, and to elaborate on the answers. A pilot interview was conducted to confirm that the order of the questions and the wording was logical and understandable.

### 2.4. Procedure of the interviews

CB (female), a medical student with experience in interview techniques, conducted the interviews. Twelve medical doctors were interviewed individually. There were no dropouts. The interviews (6 interviews at each ED conducted 1:1) were undertaken between 15th December 2021 to 31^st^ January 2022. Due to Covid-19 restrictions or work practicalities, 3 interviews were conducted digitally using Zoom. Two of these were at the hospital outside central Stockholm and 1 at the hospital in central Stockholm. The remaining 9 interviews were conducted at the hospitals where the participant worked. The interviews were digitally audio-recorded and transcribed verbatim by CB. Field notes were taken following each interview. Identifiable information in the transcripts was de-identified. Following the transcription, the informants were given the opportunity to read the transcripts, and the preliminary results were shared with the informants who were invited to read and comment.

### 2.5. Data analysis

The data collected through the interviews were analyzed through thematic analysis as outlined by Braun & Clarke.^[21]^ The data relevant for the study was analyzed separately from the rest of the data, collected through different interview questions. In total, the data generated by the 2 interview questions amounted to 16 pages of transcript. Interview transcripts were read by the 2 researchers (C.B. and A.L.). To increase the inter-rater reliability, 2 of the twelve interviews were read, discussed, and analyzed separately by C.B. and A.L. The tentative codes identified separately were then compared to see if the authors analysis coincided. The remaining interviews were coded by C.B. and reviewed by A.L. All coding was conducted in MS Word. The preliminary codes were collected and refined through discussions between the 2 researchers. The data set, organized by codes, was divided into preliminary themes and subthemes, further re-revised and agreed upon by C.B. and A.L. During the translation of codes, subthemes, themes and quotes from Swedish to English, synonyms and choice of words were continuously discussed to make translations as accurate as possible. The names of the themes, subthemes and codes were discussed in Swedish and English during the analysis to make the transition from Swedish to English language as correct as possible.

## 3. Results

### 3.1. Information about the informants

The number of years the informants had worked in the ED ranged from 3 years to ten years. The informants had finished their internship or were residents (ST-läkare) or specialists. Four of the informants were female and 8 were male (Table 1). All informants reported that their work tasks consisted of treating patients with different illnesses and conditions of varying severity, as well as making treatment plans and admitting or transferring patients.

**Table 1:** Information about the informants.

Informants	Age	Gender	Worked since	Position
1	50–55	Male	2014	Specialist
2	30–35	Male	2018	Resident
3	45–50	Male	2016	Last year resident
4	35–40	Female	2016	Last year resident
5	35–40	Male	2018	Resident
6	25–30	Male	2019	Resident
7	30–35	Female	2018	Licensed doctor
8	25–30	Male	2016	Resident
9	30–35	Male	2019	Resident
10	50–55	Male	2019	GP-specialist
11	25–30	Female	2019	Resident
12	30–35	Female	2012	Specialist

The workplace of the respondents is not specified to protect their anonymity.

### 3.2. Themes, subthemes and codes

The analysis of the interviews generated 1 theme, 4 subthemes, and 8 codes which are listed in Table 2.

**Table 2:** Themes, subthemes and codes identified in the data.

Theme	Subtheme	Code
Moral distress following patients put at risk due to communication barriers	Communication barriers causing distress for the doctor	Insecure diagnostics & risk for mistakes
	Patients vulnerable due to communication barriers	Consent and ethical dilemmas
		Difficulties in creating a safe environment
	Difficulties with translation	The doctor having little control over the conversation
		Logistical difficulties
	Insufficient resources at the ED to handle communication barriers	Extra resources needed to assess patients with communication barriers
		Taking resources from other patients
		The doctor taking shortcuts

### 3.3. Communication barriers causing distress for the doctor

#### 3.3.1. Insecure diagnostics and risk for mistakes.

Several informants reported that communication barriers caused worry about making mistakes. Further, informants reported that patients facing communication barriers were exposed to risks in the healthcare system as information could be missed, leading to mistakes and incorrect decisions. Some informants reported that barriers to communication lead to a lack of fair assessments.

“...the assessments are not fair in the same way...” Informant 3

Some informants reported that the communication barriers resulted in a risk of missing important information and making assumptions regarding the patient condition.

“...of course there is always uncertainty present—the patient could have an antibiotic allergy we don’t know about, or something similar.” Informant 4

Several informants experienced moral distress when communication barriers complicated the medical assessment. Most informants further reported that they experienced more moral distress due to communication barriers with patients with, for example, dementia, than with patients facing language barriers as language barriers can be compensated for by using an interpreter.

### 3.4. Patients vulnerable due to communication barriers

#### 3.4.1. Consent and ethical dilemmas.

Several informants reported that they experienced moral distress when assessing PLwD, as communication barriers challenge performing examinations and taking tests due to difficulties in obtaining consent from the patient. One example given referred to taking a Covid-19 PCR sample.

“If you can’t communicate with them—specifically patients with dementia who you can´t understand, and you can´t explain if you need to do something—then I can absolutely experience moral distress. It can be hard to decide what the right thing to do is, so to speak. Forcing a patient with dementia—or semi-forcing someone—to do something that they object to but that you still know is good for them.” Informant 1

Some informants described situations when they had forced patients to participate in examinations, believing it was in the patient best interest, even though it was distressing for the patient.

“They don´t understand what´s happening and it´s not really possible to reach them, and it becomes very difficult because they don´t understand the situation and what´s happening. When that is the case, and we do something that we think is necessary, they usually get scared. Sometimes they fight back, and it feels wrong - it feels a bit like coercion, and there a fine line between what okay to do and what not.” Informant 8

The informants reported that the difficulty in obtaining consent from PLwD was further complicated by the patient inability to speak for themselves.

#### 3.4.2. Difficulties in creating a safe environment.

Several informants reported that communication barriers made it difficult to create a safe, patient centered and consensual environment for their patients.

“...it becomes more ‘veterinary-medical-ish’. You treat what you have to treat, and you don’t care as much if they understand or if they feel good about it.” Informant 5

Some informants reported that they experienced that their patients suffered from their inability to communicate with the doctor.

“...I feel that they (the patients) feel very bad about it. That they often have a lot of symptoms that they have difficulty in communicating to me, and for which they cannot get proper support and symptom management.” Informant 4

Informants further reported that communication barriers were a barrier to shared decision-making.

“...There´s no guarantee that I can communicate to the patient what the (care) plan is. And that´s very sad.” Informant 4

### 3.5. Difficulties with translation

#### 3.5.1. The doctor having little control over the conversation.

Several informants reported that the translation process often contained difficult elements such as changing information, both when using an interpreter and when relatives translated.

“...as soon as there is any type of subjectivity in the interpretation, there is a risk for misinterpretation, and you can’t know for sure if your assessment is correct, and you get stomach-ache.” Informant 2

Some informants reported that they experienced lack of control when using an interpreter, as they could not be sure exactly what was communicated.

“... it can be difficult to know and difficult to have control over the situation.” Informant 3

#### 3.5.2. Logistical difficulties.

Some informants reported that assessing patients with communication barriers was time-consuming and inconvenient, and that including an interpreter often was complicated.

“You may have planned to have an interpreter time in fifteen minutes, and during that time you can simply have other things to do. So, there are many problems associated with the interpreter call itself.” Informant 3

### 3.6. Insufficient resources at the ED to handle communication barriers

#### 3.6.1. Extra resources needed to assess patients with communication barriers.

Several informants reported that assessing patients with communication barriers required more resources and was more time consuming, as more thorough clinical assessment and examination were needed, to cover up for possible mistakes and to ease the doctor worry.

“... with patients with dementia, or if there is a language barrier and I can’t get an interpreter and I can’t communicate, then I do - and I do it out of anxiety - I over-examine the patient... Which of course is time-consuming, but I´d rather do that than miss something.” Informant 2

Some informants reported that this collided with the limited resources and often stressful environment in the ED.

#### 3.6.2. Taking resources from other patients.

Some informants reported that the increase in time needed to assess patients with communication barriers affected their work situation and other patients, as the doctors could spend less time assessing their other patients, causing moral distress.

“Each such patient takes more time. Above all, it affects the total workload and the total amount of moral distress during my work shift.” Informant 3

“The moral distress [I experience] is that if I stop and talk to this patient so that I actually understand, it might delay someone else treatment.” Informant 10

#### 3.6.3. Taking shortcuts.

Some informants reported that they take shortcuts during stressful shifts, and risks in the clinical assessment, and that they are not as thorough when assessing patients with communication barriers.

“...if you’re generally stressed, you might take shortcuts and think ‘I can manage without an interpreter’......in retrospect, you realize that it wasn’t the optimal solution, but it was the simple solution - for me, not for the patient......You take more shortcuts when there is language confusion, for better or for worse.” Informant 9

## 4. Discussion

The aim of this study was to investigate doctors’ experiences of patient encounters involving communication barriers in EDs, with a focus on their experience of moral distress associated with these encounters. The analysis generated 1 main theme, 4 subthemes and 8 codes. Informants experienced moral distress due to patients being put at risk following communication barriers, caused by lack of resources and difficulties in obtaining consent. The doctors further experienced that using an interpreter was associated with feelings of lack of control, and logistical difficulties. Moreover, the doctors experienced moral distress due to patients with communication barriers needing more resources, leaving less resources to other patients in the ED, and due to lacking consent when assessing the patient.

Informants experienced increased moral distress when assessing PLwD, since the communication barriers were not able to compensate for by using an interpreter, as opposed to language barriers. There were different opinions on to what extent assessing patients with language barriers affected levels of moral distress. There were also varying opinions on what extent the use of an interpreter makes the encounter more complicated. In accordance with some informants’ doubt whether accurate translation when using an interpreter, an interdisciplinary Belgian study found that accurate interpretation occurred in as few as 19% of interpreter speech turns. The study found through previously used scores on clinical significance and definitions of communication tasks that inaccurate interpretation had a clinical impact, and that the greatest impact was caused by the interpreter answering for the patient. The study further found that interpretation negatively affected the doctor-patient relationship, which was mostly impacted by the interpreter omitting messages.^[17]^

Instead of using an interpreter, some bilingual informants would, if possible, communicate directly with the patients in their native language. Informants further reported that it was not uncommon to use relatives for translation. Previous studies have found that bilingual healthcare workers as well as relatives are commonly used for translation in EDs and other healthcare settings instead of professional interpreters.^[22,23]^ Using relatives for translation has however been found to be error-prone.^[24]^ Further, the usage of relatives, especially younger children, can be problematic from both the carer perspective and relative perspective.^[23,25]^ Patients not having access to an interpreter can also result in extensive mistakes. For instance, in a case in California, a healthy kidney was removed instead of the diseased one, as a patient underwent a nephrectomy after the patient signed a consent form in English and was not provided with an interpreter.^[26]^ Relevant from the Swedish perspective is that while the need for interpreters in Swedish healthcare has increased with a growing percentage of foreign-born in Sweden,^[14,27]^ the Swedish government has recently suggested that the patient should pay for translation services when interpretation is needed in healthcare.^[28]^

In accordance with the informants worry regarding making incorrect clinical judgements and assessments, previous research has found that dementia indeed is associated with decreased accuracy of the older patient presenting illness,^[29]^ and that communication barriers pose risks for patients.^[13,15–18]^ Specifically for PLwD, whose ED visits are associated with adverse outcomes including functional decline, hospitalization, and mortality,^[3,10,11,30,31]^ with one of the found reasons for adverse outcomes being communication barriers.^[11]^ Indeed, PLwD are less likely to comprehend their discharge diagnosis and instructions.^[29]^ ED visits for PLwD are further associated with higher mortality and higher costs compared to patients without dementia,^[3,32]^ in line with the informants’ experience of PLwD requiring more resources. Nevertheless, PLwD are often excluded from research on communication and shared-decision-making in emergency departments and evidence-based strategies to improve ED communication for PLwD do not yet exist.^[8]^ To develop such strategies which are appropriate and feasible, more qualitative research on healthcare workers experience of assessing patients with communication barriers is needed. One of few previous studies that have been conducted on doctors´experience of assessing patients with communication barriers found that caregiver presence can have an important role.^[33]^ A qualitative study on language barriers involving both patients and healthcare workers suggested more access to interpreters as a measure.^[13]^ Another article on language barriers found that bedside access to a phone interpreter improved patient reported informed consent. The authors further suggested additional clinician educational interventions.^[34]^ Taking measures to improve quality of care in general and adapt clinical instruments to PLwD is also crucial. One example of such is the translation and cultural adaptation of the Abbey Pain Scale.^[35]^ Also, suggestions on how to limit ED visits for PLwD.^[36]^ Although this study sheds light on the facts that communication barriers can contribute to moral distress, adding to a broader understanding of the concept, there is a lack of evidence-based methods to manage moral distress. The results of this study can be considered when developing such methods.

Limitations to this study include that the interview questions were asked as part of a questionnaire focusing on the experiences of moral distress during the pandemic. The different focus might have affected the informants’ way of considering the questions and the relevance of their experience of moral distress related to communication barriers, unrelated to the Covid-19 pandemic. The sample size can also be considered as relatively small. However, rich findings can in fact be discovered with small datasets, as stated by Young et al.^[37]^ Data was further collected until saturation was reached as deemed by Urquhart^[20]^ which is a strength. Another relevant aspect to consider is that only 2 questions about language and communication barriers were asked, providing little data. The questions’ focus on language and communication barriers combined can further be seen as a limitation. If the interview had focused entirely on language or communication barriers instead of having an overarching focus on moral distress during the Covid-19 pandemic, more aspects and in-depth data would probably have been generated. However, the nature of the study entails that generalization might be a problem. The fact that 2 questions were asked also allowed for discussion on differences between moral distress due to language barriers and communication barriers. A strength of the study is that principal researcher CB works clinically, providing understanding of the context and situations that the participants presented.

## 5. Conclusions

In conclusion, informants experienced moral distress when assessing patients with communication barriers due to difficulties in mediating calm and safety to their patients. Also due to difficulties in obtaining consent before conducting examinations and interventions. As informants could not obtain proper information from their patients, worry of making mistakes increased. Informants described that communication barriers resulted in patients needing an unnecessarily thorough clinical assessment and evaluation, taking time and resources from other patients, and causing moral distress. Although communication through an interpreter was associated with inconvenience and lack of control, the results suggest that informants experienced increased moral distress when assessing patients with communication barriers due to dementia and disabilities, compared to language barriers, as these could be compensated for through interpreters. The results suggest that communication barriers can be a cause of moral distress for doctors in emergency departments, which should be considered when developing tools and methods to mitigate and manage moral distress. More research is needed on which communication strategies to use when assessing PLwD.

## Acknowledgments

We would like to thank the informants. We are very grateful for their time and participation.

## Author contributions

**Conceptualization:** Clara Brune, Ann Liljas.

**Data curation:** Clara Brune.

**Formal analysis:** Clara Brune.

**Funding acquisition:** Clara Brune.

**Investigation:** Clara Brune.

**Methodology:** Clara Brune, Ann Liljas.

**Project administration:** Ann Liljas.

**Resources:** Clara Brune, Ann Liljas.

**Software:** Clara Brune.

**Supervision:** Ann Liljas.

**Validation:** Clara Brune, Ann Liljas.

**Visualization:** Clara Brune.

**Writing – original draft:** Clara Brune.

**Writing – review & editing:** Clara Brune, Ann Liljas.
